# Genomic copy number variability at the genus, species and population levels impacts in situ ecological analyses of dinoflagellates and harmful algal blooms

**DOI:** 10.1038/s43705-023-00274-0

**Published:** 2023-07-08

**Authors:** Rendy Ruvindy, Abanti Barua, Christopher J. S. Bolch, Chowdhury Sarowar, Henna Savela, Shauna A. Murray

**Affiliations:** 1 University of Technology Sydney, School of Life Sciences, Sydney, PO Box 123, Broadway, NSW 2007 Australia; 2 Institute for Marine & Antarctic Studies, University of Tasmania, Launceston, 7248 TAS Australia; 3 Sydney Institute of Marine Science, Chowder Bay Rd, Mosman, NSW Australia; 4 Finnish Environment Institute, Marine Research Centre, Helsinki, Finland

**Keywords:** Molecular ecology, Molecular evolution

## Abstract

The application of meta-barcoding, qPCR, and metagenomics to aquatic eukaryotic microbial communities requires knowledge of genomic copy number variability (CNV). CNV may be particularly relevant to functional genes, impacting dosage and expression, yet little is known of the scale and role of CNV in microbial eukaryotes. Here, we quantify CNV of rRNA and a gene involved in Paralytic Shellfish Toxin (PST) synthesis (*sxtA4*), in 51 strains of 4 *Alexandrium* (Dinophyceae) species. Genomes varied up to threefold within species and ~7-fold amongst species, with the largest (*A. pacificum*, 130 ± 1.3 pg cell^−1^ /~127 Gbp) in the largest size category of any eukaryote. Genomic copy numbers (GCN) of rRNA varied by 6 orders of magnitude amongst *Alexandrium* (10^2^– 10^8^ copies cell^−1^) and were significantly related to genome size. Within the population CNV of rRNA was 2 orders of magnitude (10^5^ – 10^7^ cell^−1^) in 15 isolates from one population, demonstrating that quantitative data based on rRNA genes needs considerable caution in interpretation, even if validated against locally isolated strains. Despite up to 30 years in laboratory culture, rRNA CNV and genome size variability were not correlated with time in culture. Cell volume was only weakly associated with rRNA GCN (20–22% variance explained across dinoflagellates, 4% in Gonyaulacales). GCN of *sxtA4* varied from 0–10^2^ copies cell^−1^, was significantly related to PSTs (ng cell^−1^), displaying a gene dosage effect modulating PST production. Our data indicate that in dinoflagellates, a major marine eukaryotic group, low-copy functional genes are more reliable and informative targets for quantification of ecological processes than unstable rRNA genes.

## Introduction

Genomic copy number variation (CNV) is increasingly documented in eukaryotic, bacterial and archaeal genomes [1–5], and represents a major source of intra-specific and population-level genetic variation. The impact of CNV on phenotypic trait expression has been characterised in flowering plants, vertebrates, yeast, and human health research including many model organisms [1–5]. Eukaryote CNVs can lead to increased expression and dosage, providing a potential selective advantage [1, 3, 5–7]. Despite its potential importance, the scale and role of CNV in most non-model organisms, including marine microbial eukaryotes, is poorly understood.

While CNV has been reported in marine microbial eukaryotes [8–13], and a few studies have indicated rRNA genes could vary in copy numbers or sequences [14, 15], it is still relatively unclear whether CNV has a significant impact on quantitative molecular ecological studies employing meta-barcoding, meta-genomics and qPCR [10]. Quantitative molecular ecology studies of marine protists generally use regions of the rRNA operon for community structure analyses due to the broad coverage of rRNA genes in reference databases, the capacity to resolve taxa, and a high genomic copy number (GCN) in eukaryotes (>10^2^ cell^−1^) which aids in the detection sensitivity [9–12]. However, in animals, fungi and plants, rRNA gene copies are variably present, from 10^2^–10^4^ copies cell^−1^ [9, 10, 16–18], with a similar range (10^3^–10^4^ copies cell^−1^) in diatoms (Stramenopiles) [19]. Other groups of microbial eukaryotes may show greater variation, from 10^3^- 10^5^ copies cell^−1^ in ciliates (Alveolata) [11], and 10^2^–10^5^ in foraminifera (Rhizaria) [12]. Within most species of microbial eukaryotes, rRNA gene copy numbers are considered to be more stable [19, 20], however, relatively few studies have examined this [11, 12, 19].

Dinoflagellates encompass most harmful algal bloom (HAB) forming taxa, as well as constituting up to 50% of marine microbial eukaryotic biomass, thus are a major constituent of aquatic microbial ecosystems [13]. Genome size varies considerably in dinoflagellates (~1 Gb to >150 Gb) including some of the largest known eukaryotic genomes, larger than the largest animal (lungfish, 130 Gb) and plant (*Paris japonica*, 149 Gb) genomes [21–25]. Gene duplication and large-scale expansion appear to have occurred amongst dinoflagellate genomes, and coding genes are often present in multiple tandem repeats [26–30]. Genomes of coral symbiont species (Dinophyceae: Symbiodinaceae) show highly dynamic evolution, driven by gene family expansion via both tandem duplication [28, 29] and retroposition [31, 32]. Considerable genome size variation and very large genomes occur in multiple planktonic dinoflagellate orders [33], as well as in other groups of marine microbial eukaryotes such as foraminifera, ciliates, and Amoebozoa [33]. GCN of rRNA genes across much of eukaryotic life are considered broadly correlated with genome size [18]. Such large and dynamic genome sizes suggest substantial CNV may exist in these taxa.

Of marine harmful algal blooms forming taxa, those that produce Paralytic Shellfish Toxins (PSTs) are common and have significant public health and economic implications [34]. PST expression can constitute an inducible defence mechanism in marine dinoflagellates in response to the presence of copepod predators [35]. PSTs are synthesised by the cosmopolitan and common marine dinoflagellates *Alexandrium* species, *Pyrodinium bahamense*, *Gymnodinium catenatum*, and *Centrodinum punctatum*. Dinoflagellate genes associated with PST biosynthesis (*sxt*) [36–38] possess dinoflagellate features such as a unique 22 bp spliced leader sequence on transcripts, a high GC-content, and eukaryotic poly-A tails [36, 38]. A relatively low proportion of genes (~10–27%) in dinoflagellates are thought to be regulated at the transcriptional level [21, 23, 26], with many genes regulated post-transcriptionally. The role of gene dosage acting on this trait may therefore differ in dinoflagellates from that in more highly transcriptionally regulated taxa. Studies of certain species such as *A. minutum* and *A. ostenfeldii* have indicated a correlation may exist between cellular PST content and genomic copies of the PST biosynthetic gene *sxtA4* [39–41]. Some studies have shown that PST synthesis may not be regulated at the transcriptional level [42, 43]. This gene has been found to vary in GCN across studies, as *A. pacificum* and *A. catenella* show ~180–325 copies cell^−1^ of *sxtA4* [40, 44, 45]; while *A. minutum* and *A. ostenfeldii* showed fewer copies, at 1.5–11 copies cell^−1^ [39, 41].The majority of species of *Alexandrium* had no detectable *sxtA4* copies and do not produce PSTs [46]. Thus *sxtA4* is a gene with a comparatively lower copy number in dinoflagellates than those that show large scale tandem repeats [26–30]. If consistent across PST-producing species, GCN may constitute a useful marker for *in situ* ecological analyses of HABs, and potentially for other functional traits governed by genomic dosage.

Because rRNA genes, as compared to coding genes, are likely to be under different selective pressures [47], processes that lead to CNV may differ between them. To determine the impact of CNV on both a functional gene and rRNA barcoding markers, and to examine the role of genome size and time in culture on CNV, we quantified CNV of rRNA genes and *sxtA4* in relation to genome size across 51 strains of PST-producing marine dinoflagellate, *Alexandrium australiense, A. pacificum, A. catenella* and *A. minutum*. Our selection of strains provided capacity to examine CNV within and between species, in strains maintained in long-term culture, and CNV variance across regions. As diversity analysis employing rRNA genes in particular becomes ubiquitous, we aimed to determine the scale of biases associated with CNV, examine its prevalence across dinoflagellates and indicate potential solutions.

## Materials and methods

### Culture isolation, maintenance and identification

Fifteen non-axenic strains of *Alexandrium pacificum* were established from a surface net haul collected on 22/11/18 at Mindarie Marina, Western Australia (−31.689127, 115.703103). Single cell isolation of *A. pacificum* was performed using drawn out glass pipettes and a Nikon Eclipse TS100 inverted microscope (100x magnification). Isolated cells were transferred into Falcon®24 well culture plates containing 1 ml of K/5 medium [48] without sodium silicate. Germanium dioxide was added (5 µg/ml) to prevent diatom growth. Plates were kept at 18 °C under a photon flux of 60–100 μmol photons PAR m^−2^ s ^−1^ with a 12/12 h dark/light cycle (cool white fluorescent). After 3 weeks, the cultures were transferred into 20 ml K media in 70 mL sterile culture flasks (Thermo Fisher Scientific, Massachusetts, USA), and maintained by serial transfer every 3 weeks. In total 36 additional strains of 4 *Alexandrium* species (*Alexandrium catenella, A. minutum, A. pacificum, A. australiense*; Supplementary Table 1) were obtained from collections: the Australian National Algae Culture Collection (CS), the Cawthon Institute Culture Collection of Microalgae (CAWD), the Roscoff Culture Collection (RCC), and collections maintained at the Institute for Marine and Antarctic Studies, University of Tasmania. Strains originated from 8 different countries in Europe, Asia, Australasia and the Americas, across states and regions in Australia, and were isolated on differing dates within the past 30 years.

Isolate identity was confirmed by sequencing the D1-D3 region of large-subunit rRNA. Cells were harvested from 50 ml of culture by centrifugation and DNA extracted using the FastDNA spin kit for soil (MP Biomedicals, Santa Ana, California, USA). DNA quality was checked using a Nanodrop 2000 (Thermo Scientific, Waltham, Massachusetts) spectrophotometer. The D1-D3 region of LSU-rRNA was amplified by PCR using primers D1F [49] and D3B [50] in 25 µl reactions containing 5 µl of 5X MyTaq buffer (Bioline, London, UK), MyTaq polymerase (Bioline, London, UK) 0.5 µl, 7.5 pmol of each primer, 1 µg µl^−1^ BSA (Biolabs, Arundel, Australia), 1 µl of DNA and 15.5 µl of DNA-free water. PCR conditions were 94 °C for 5 min, followed by 35 cycles of: 94 °C for 30 s, 56 °C for 30 s, and 72 °C for 1 min; and a final extension step of 3 min. PCR products were verified by 1% agarose gel electrophoresis stained with GelRed (Gene Target Solutions, Dural, Australia) and purified with Zymoclean™ (Zymo Research, California, USA). Sanger sequencing of products was performed by Macrogen (South Korea), with strains assigned to *A. pacificum, A. catenella, A. minutum* or *A. australiense* based on comparisons with sequences from verified isolates of each species.

### Culture synchronisation and harvest

To measure genome size, CNV and PSTs, *Alexandrium* spp. were grown to exponential phase in GSe medium [51] at 18 °C. For genome size measurement of cell cycle synchronised strains, cells were then incubated for 48 h in darkness to induce synchronisation of cell division [52, 53]. Sub-samples were fixed with Lugol’s iodine at the point of harvest, and cell concentration determined using a Sedgewick-Rafter counting chamber (ProSciTech, Australia) and an inverted light microscope (Leica Microsystems, Wetzlar, Germany). For CNV quantification with qPCR, ~60–75 × 10^3^ cells in triplicate from each strain were harvested by centrifugation (10 min at 1000 g). For genome size quantification using flow cytometry, ~10^5^ cells were harvested in triplicate from each strain. At least 10^6^ cells were harvested by centrifugation for 10 min at 1000 g for PST measurement.

### Genome size measurement

Cells were washed with a 1 × PBS, fixed with 1% (w/v) paraformaldehyde for 10 min, and then washed again with 1 × PBS. Cell pellets were re-suspended in 2 mL cold methanol, stored for at least 12 h at 4 °C to remove the intracellular chlorophyll, washed twice with 1x PBS, and then stained for > 3 h in 0.1 mg mL^−1^ propidium iodide and 2 µg mL^−1^ RNAse (Merck KGaA, Darmstadt, Germany).

A CytoFLEX S Flow cytometer (Beckman Coulter, California, USA) equipped with laser excitation at 488 nm was used for the flow cytometry analysis. BD™ DNA QC Particles Chicken blood cells (3 pg DNA/nuclei; BD Biosciences, San Jose, USA) were used as a standard [54]. In total, 2-µm fluorescent beads were used as stable particles to verify instrument alignment (BD Biosciences, San Jose, USA). Triplicate samples were run at 30 µL min^−1^, and data acquired in linear and log modes until at least 10,000 events were measured per sample. Fluorescence emission of propidium iodide stained DNA was detected at 610 ± 10 nm. The peak ratios and coefficient of variation (CV) were quantified with CytExpert software (Beckman Coulter, California, USA). FSC channel was used as a trigger with automatic setting from the manufacturer. Gating and further analysis were only performed for peaks with CV values below 20%, and any peaks above this were rerun. The gating was performed by using FSC-A vs SSC-A gate to exclude debris and use the PI gate on histogram to remove large background noise without the DNA content. Genome size in base pairs used a conversion factor of 1 pg of DNA = 978 Mbp [55].

### Genomic copy number quantification with qPCR

DNA extraction was carried out using a PowerSoil DNA Extraction kit (QIAGEN, OH, USA) according to manufacturer’s instructions. DNA was extracted in triplicate, and quality and quantity determined using a Nanodrop ND-1000 (ThermoFisher Scientific, Waltham, Massachusetts) and Qubit 2.0 Fluorometer (ThermoFisher Scientific, Waltham, Massachusetts). qPCR was carried out with a BioRad CFX384 Touch™ System (BioRad, California, USA) using species-specific qPCR assays for *Alexandrium* rRNA genes [56] and *sxtA4* [44] (Supplementary Table 5, Supplementary Fig. 8). qPCRs were run in triplicate using the following cycling parameters: 95 °C for 10 s, 35 replicates of 95 °C for 15 s and 60 °C for 30 s. Total reaction volume was 10 μl, containing 5 μl SybrSelect™ (ThermoFisher Scientific, Massachusetts, USA), 0.5 μM each primer, 1 μl template DNA, and 3 μl PCR-grade water. Samples and Master Mix were loaded to Hard-Shell 384-well PCR plates using epMotion 5075 Liquid Handling Workstations (Eppendorf AG, Hamburg, Germany). Amplification specificity was confirmed using melt-curve analysis. Quantification cycle values were generated by CFX Manager 3.1. Standard curves of *sxtA4* and rRNA genes vs quantification cycle (Cq) was developed using ten-fold serial dilution of Gblocks® fragments (Integrated DNA Technologies, Coralville, Iowa, USA). Copy number per µL DNA was determined using the formula:

$$copy\,number\,per\,{{{{{{{\mathrm{\mu }}}}}}}}l\,DNA = \frac{{DNA\,amount\left( {ng} \right)per{\kern 1pt} {{{{{{{\mathrm{\mu }}}}}}}}l\,x\,6.022\,x\,10^{23}}}{{length\,of\,fragment\left( {124bp} \right)x\,660\,x\,10^9}}$$

Standard curves, positive controls and negative controls were included in each sample plate as the sample DNA extracted from *Alexandrium* strains of known concentration (cells µL^−1^). Copies of *sxtA4* and rRNA genes per µL^−1^ DNA were determined relative to qPCR standard curves.

### Statistical analyses

The significance of relationships between genome size, rRNA gene copies cell^−1^, *sxtA4* copies cell^−1^ and total PST cell^−1^ were assessed using Spearman’s rank correlation and linear regression after transformation, as appropriate, as implemented in GraphPad Prism 7.04. Shapiro-Wilk tests were used to examine normal or log normal distributions. Patterns of genome size and rRNA gene CNV associated with cell culture were based on isolation dates provided by culture collections and isolators. Days from isolation date to sample extraction date were calculated. To account for different laboratory growth rates, cultivation days were converted to estimated number of generations based on published growth rates for each species at culture maintenance conditions (50–80 μmoles PAR; 12:12 L:D cycle18–20 °C). Individual strain variance from respective species means were calculated for genome size (pg) and log_10_ rRNA gene (copies cell^−1^). Individual strain deviation from species means were calculated using ([(Xi - X̅)/*σ*)]. The effect of extended culture periods on variability in genome size and rRNA gene GCN was examined using Levene’s test for equal variances among the following three sample groups: (1) Mindarie isolates cultivated for <20 generations; (2) strains cultivated for 100–800 generations; (3) strains cultivated for >1000 generations.

### PST quantification

Cell pellets were freeze-dried, then extracted for PSTs and measured [53]. Briefly, samples were resuspended in 2 mL of 1 mM acetic acid and vortexed for 90 s at 100 °C for 5 min, sonicated for 5 min and filtered with 0.45 µm PVDF filter (Merck Millipore, Massachusetts, USA). Chromatographic separation (modified [57]) was performed on a Thermo Scientific™ ACCELA™ UPLC system coupled to a Thermo Scientific™ Q Exactive™ (ThermoFisher Scientific, Massachusetts, USA) mass spectrometer. Analysis was performed using an Acquity UPLC BEH Amide 130 A 1.7 µm 150 × 2.1 mm column with an injection volume of 5 µL. Mobile phases were A1 (water/formic acid/NH4OH at 500:0.075:0.3 v/v/v), B1 (acetonitrile/water/formic acid at 700:300:0.1 v/v/v). Certified standard solutions of C1,C2, GTX1, GTX2, GTX3, GTX4, GTX5, dcGTX2, dcGTX3, STX, dcSTX, NEO and dcNEO were purchased from National Research Council of Canada (NRC, Halifax, Canada). The limit of detection of the PST analysis method for all of the targeted compounds was 0.01 pg cell^−1^.

## Results

### Genome size

**Fig. 1:**
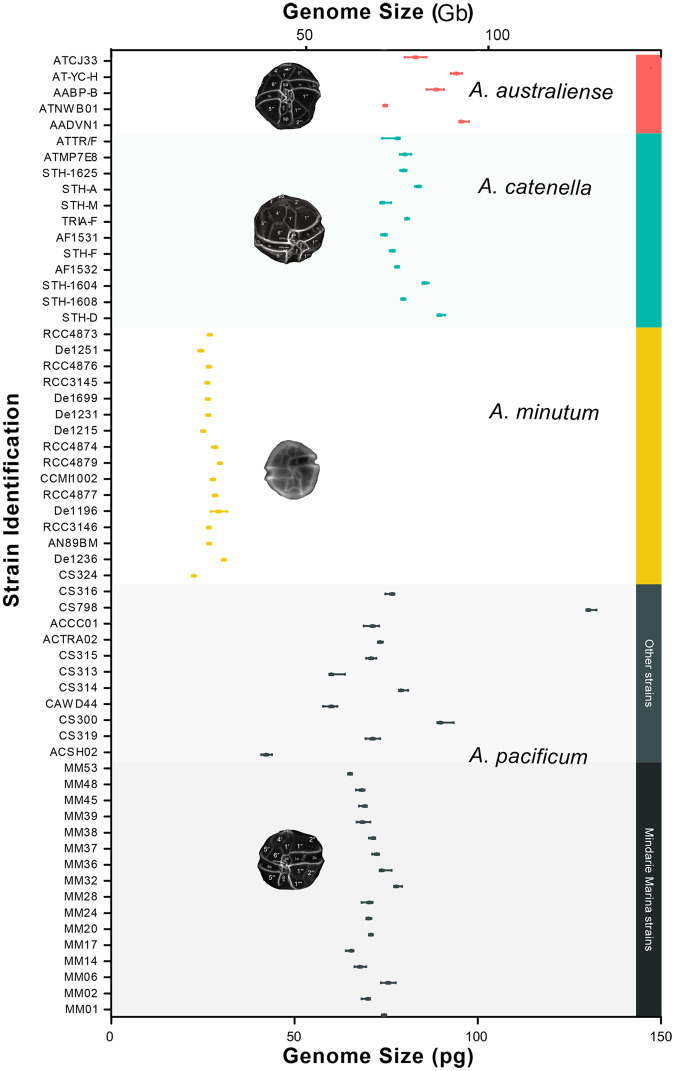
Genome sizes (pg cell^−1^, Gb cell^−1^) of strains from four dinoflagellate species (Gonyaulacales: *Alexandrium*). Images show cells using fluorescence light microscopy, stained with Calcofluor white, from [83, 84].

**Fig. 2:**
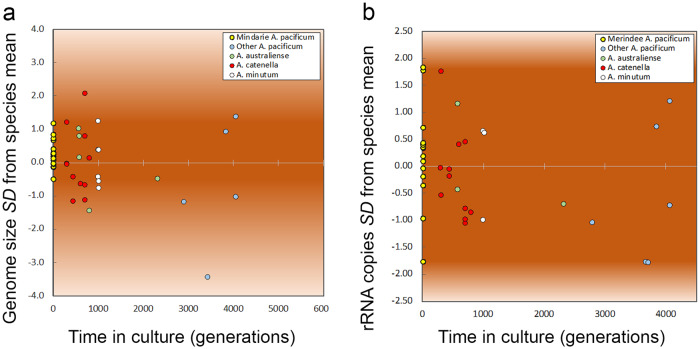
Variation of genome size and rRNA copy number of *Alexandrium* species with generations in laboratory culture. **a** Variation in *Alexandrium* spp genome size with generations in laboratory culture. Values are *SD* from the mean genome size. Darker background shading indicates range of deviation among Mindarie (WA) *Alexandrium pacificum* strains cultured for <20 generations prior to analysis. **b** Variation in *Alexandrium* species rRNA copy number with increasing generations in laboratory culture. Values are *SD* from the mean rRNA copy number of each species. Darker background shading indicates range of deviation among Mindarie (WA) *Alexandrium pacificum* strains cultured for <20 generations prior to analysis.

Genome sizes of all species were large, and varied greatly between species, from 22.5 ± 0.3 pg cell^−1^ to 130.9 ± 1.3 pg cell^−1^ (Fig. 1). The genome of *A. minutum* was the smallest (27.0 ± 2.0 pg cell^−1^, *n* = 16) while species of the *A.tamarense* species complex were much larger: *A. catenella* (79.2 ± 5.9 pg cell^−1^, *n* = 13), *A. pacificum* (72.5 ± 14 pg cell^−1^, *n* = 27) and *A. australiense* (87.3 ± 8.2 pg cell^−1^, *n* = 5). Strains of *A. pacificum* showed more than threefold variation in genome size (42.4 ± 1.5 pg cell^−1^ to 130.9 ± 1.3 pg cell^−1^) (Fig. 1). Genome size among *A. pacificum* strains from the same population varied by 13% above and 9% below the population mean (CV = 5.1%, Fig. 1). *A. pacificum* genomes from different populations, different global regions, and time in laboratory culture varied over a wider range (CV = 29.5%, Fig. 1), however, there was no clear relationship between genome size and time in laboratory culture, or significant increase or decrease in genome size variability over time in culture (Fig. 2a, Supplementary Fig. 1A, F = 2.331, df=2, *p* = 0.110).

### CNV of rRNA gene and *sxtA4* among and within species

**Fig. 3:**
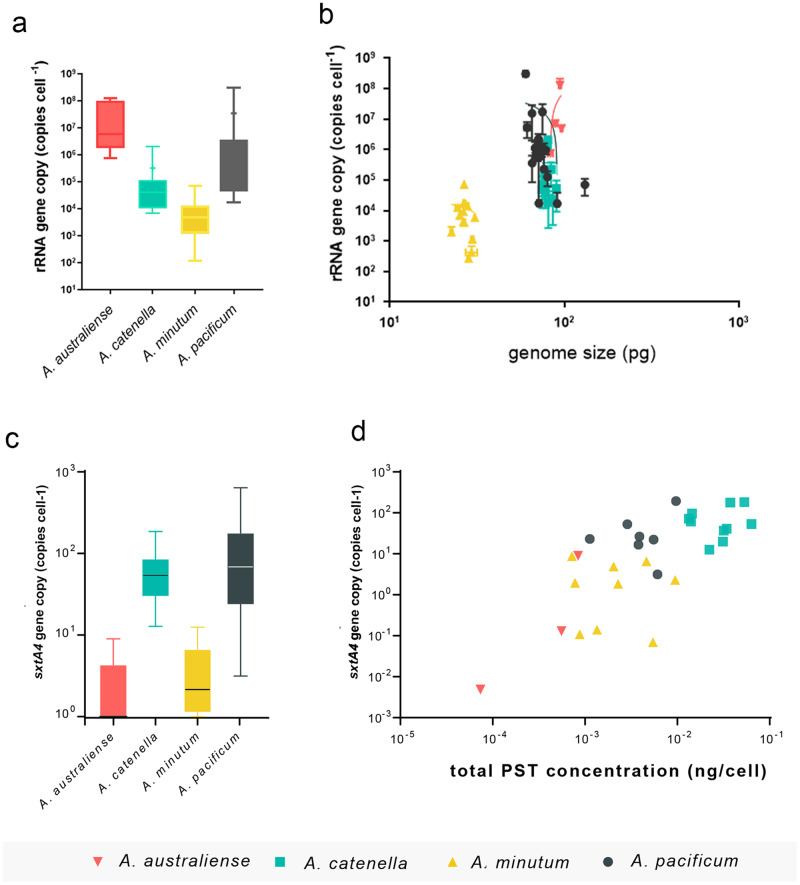
The relationship between the CNV of marker genes and the genome size and total PSTs in *Alexandrium* species. **a** CNV of rRNA (±SD) in strains of *Alexandrium spp*. **b** Relationship between genome size (pg cell^−1^) and rRNA copies cell^−1^ in *Alexandrium* species (F = 22.99, df=49, *p* < 0.0001, r^2^ = 0.319). **c** CNV of *sxtA4* (±SD) in *Alexandrium* species. **d** Relationship between total PSTs (ng cell^−1^) and s*xtA4* copies cell^−1^ in *Alexandrium* species (F = 11.73, df=18, *p* = 0.003, r^2^ = 0.395).

**Fig. 4:**
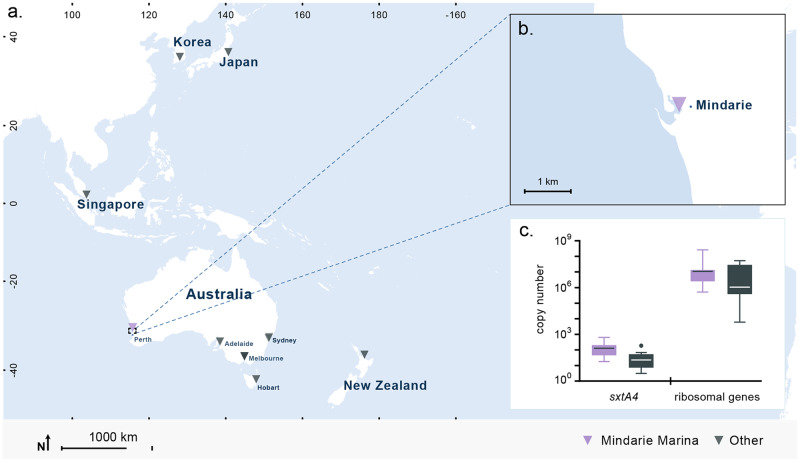
Copy number variations comparison between *A. pacificum* isolated from a bloom site against strains from other sites. **a** Sites where strains of *A. pacificum* were isolated from the Pacific region including sites across Australia. **b** Mindarie from where strains of *A. pacificum* were isolated. **c** CNV of *sxtA4* (±SD) and rRNA (±SD) in *Alexandrium pacificum* from all sites and from Mindarie only.

rRNA GCN varied by 6 orders of magnitude across *Alexandrium* species, from 267 ± 18.8 copies cell^−1^ in a strain of *A. minutum* (RCC4877) to 1.23 × 10^8^ ± 85 × 10^6^ copies cell^−1^ in a strain of *A. australiense* (AT-YC-H) (Fig. 3a). In general, strains of *A. australiense* and *A. pacificum* showed the largest rRNA GCN, while those of *A.minutum* were the smallest and most uniform (Fig. 3a). Within-species CNV of rRNA genes was surprisingly high, particularly among isolates of *A. pacificum*, which varied from 1.74 × 10^4^ ± 1.26 × 10^3^ copies cell^−1^ to 1.72 × 10^7^ ± 1.40 × 10^7^ copies cell^−1^, and more than two orders magnitude (8.1 × 10^4^ ± 1.40 × 10^4^ copies cell^−1^ to 1.72 × 10^7^ ± 1.41 × 10^7^ copies cell^−1^) in a single population (Fig. 4). Increased generations in laboratory culture (Fig. 2b, Supplementary Fig. 1B) had no effect on mean rRNA GCN or CNV of *Alexandrium* species (F = 1.819, df=2, *p* = 0.177). Variability was evident in *sxtA4* copies across *Alexandrium* species, though at a smaller scale than in rRNA gene (Fig. 3c, Supplementary Fig. 2). The *sxtA4* GCN of *A. pacificum* strains showed the greatest variability, from 7.8 ± 1.9 copies cell^−1^ to 609 ± 133 copies cell^−1^, but were more uniform among strains from the same population (43.8 ± 9 copies cell^−1^ - 609 ± 133 copies cell^−1^), while the variability of *sxtA4* GCN in *A. minutum* and *A. australiense* strains were low, from 0–8.9 ± 0.61 copies cell^−1^ (Fig. 3c). *A. catenella* strains varied from 19.9 ± 10.5 copies cell^−1^ - 189.12 ± 58.5 copies cell^−1^ (Fig. 3c). A significant positive relationship between log_10_ rRNA copies cell^−1^ and genome size was evident (Fig. 3b, F = 22.99, df=49, *p* < 0.0001, r^2^ = 0.319) across the species of *Alexandrium*, however at the species level this was only significant within *A. pacificum* (F = 6.7, df=18, *p* = 0.0185, r^2^ = 0.27). A significantly non-zero slope was found in the relationship between log_10_ *sxtA4* copies cell^−1^ and genome size but explaining only a very low amount of the variance (Supplementary Fig. 2A, F = 5.3, df=43, *p* = 0.026, r^2^ = 0.109).

### PST content and its relationship to *sxtA4* copy number

**Fig. 5:**
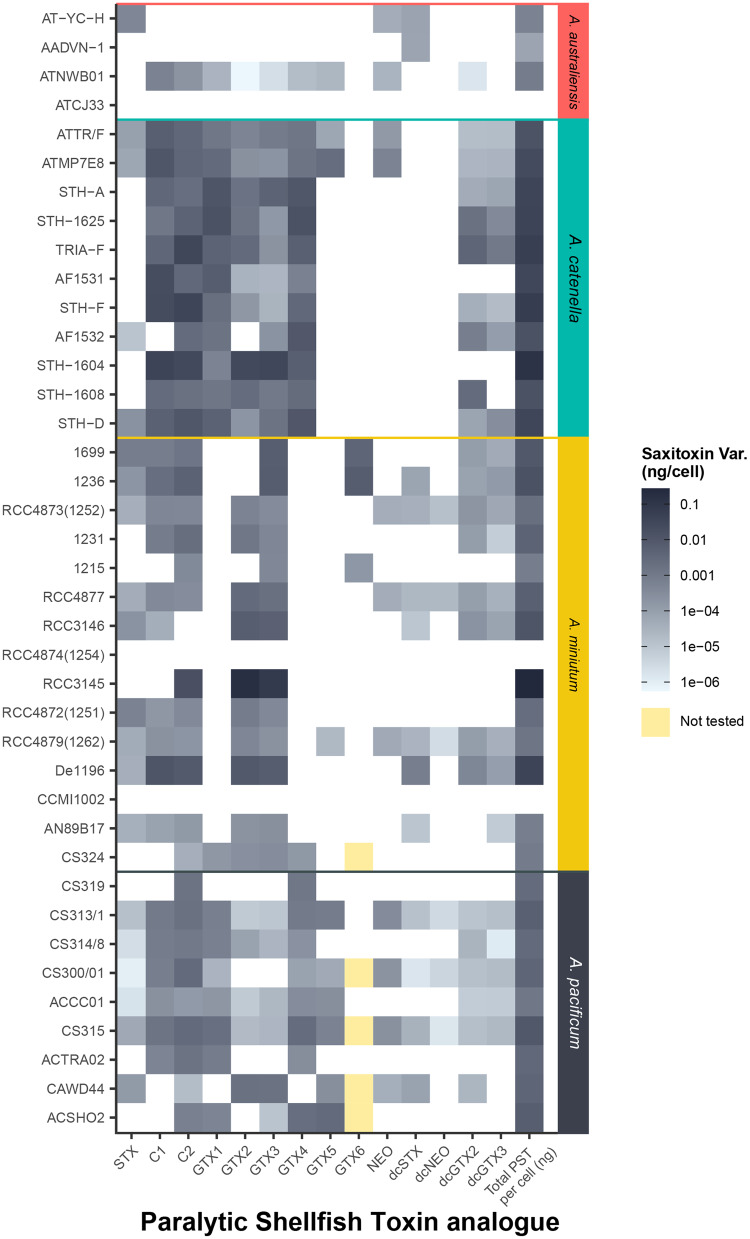
Concentration of individual PST congeners (ng PST cell^−1^) in *Alexandrium* species. The heatmap shows the inter-species and intra-species variations of each PST congeners' concentration across *Alexandrium* spp.

All strains produced PSTs with the exception of *A. australiense* ATCJ33 and two strains of *A. minutum*, RC4874 and CCMI1002. The range of PST congeners produced varied considerably between and within species (Fig. 5). Species of *A. catenella* contained the highest PST content (Figs. 5, 3d) dominated by C1, 2 and GTX1, GTX2, GTX3 and GTX4. Both GTX5, GTX6, as well as decarboxylated versions of STX and NeoSTX were mostly absent. In contrast, *A. minutum* strains produced a more restricted range of PSTs, with C2, GTX2 and GTX3 produced (Fig. 5). Total PSTs were more uniform among *A. pacificum*, with no consistently dominant PST variants. A positive relationship was significant between log_10_ *sxtA4* copies cell^−1^ and log_10_ total PSTs ng cell^−1^ (Fig. 3d, F = 11.73, df=18, *p* = 0.003, r^2^ = 0.395). *A. minutum* produced a higher amount of PST per copy of *sxtA*4 in comparison to *A. pacificum*. Strains of *A. australiense* with extremely low or no detectable PSTs (ATCJ33, AADVN1, AT-YC-H), averaged <1 *sxtA4* copy cell^−1^. In cases where less than one *sxtA4* copy cell^−1^ was found, this was due to copy numbers below the level of quantification of the qPCR assay.

## Discussion

### CNV in microbial eukaryotes and its functional significance

Genomic CNV in eukaryotes, bacteria and archaea has been increasingly documented, sometimes in relation to whole genome duplication or polyploidy [1–5]. CNV is considered a major evolutionary process, influencing the expression of key phenotypic traits [1, 2, 4–7]. The presence of CNV is also of practical significance, impacting the way in which molecular barcoding genes such as rRNA, commonly used for community structure analyses, can be interpreted. The consistency of GCN is poorly understood in microbial eukaryotes, and the causes and consequences of CNV at the genomic level [2, 4, 5] have been rarely examined.

**Fig. 6:**
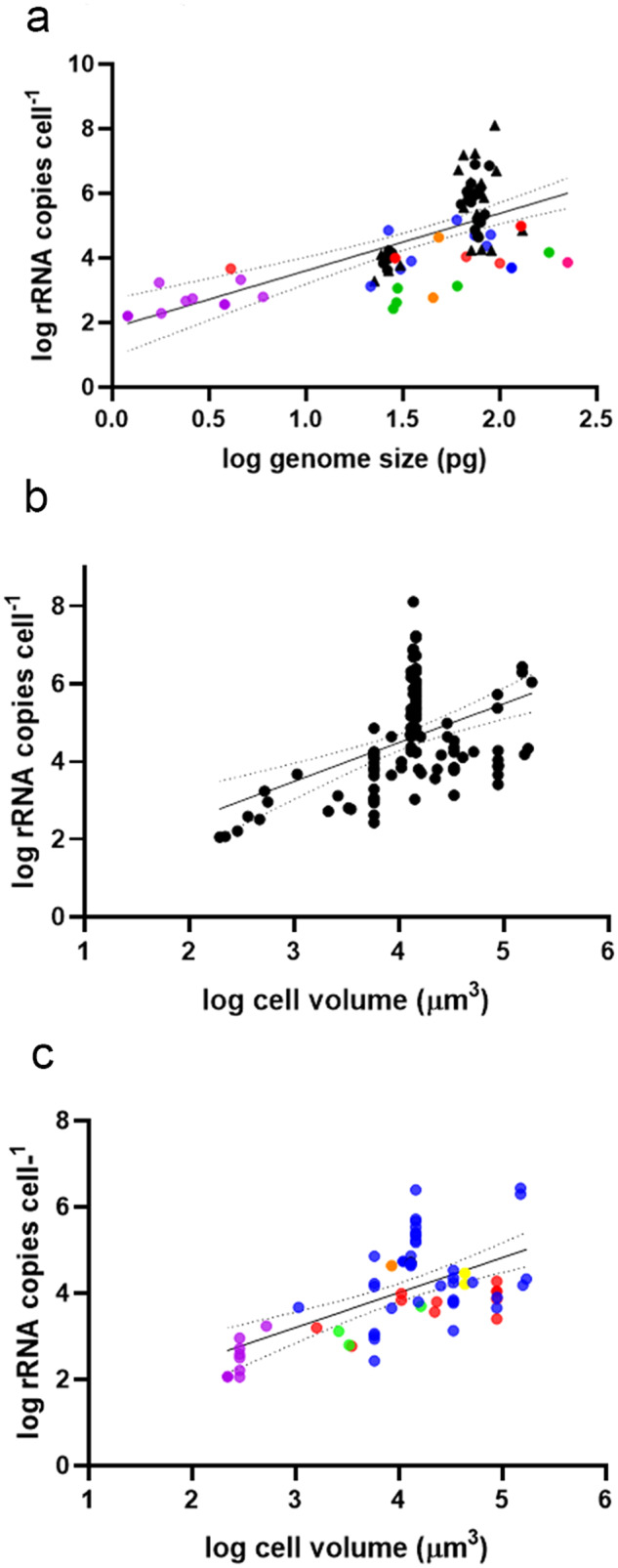
Relationship between rRNA copies cell^−1^ and the genome size and cell volume in dinoflagellates. **a** Relationship between log_10_ genome size (pg cell^−1^) and log_10_ rRNA copies cell^−1^ in dinoflagellates from the current study and previous literature (F = 44.2, df=74, *p* < 0.0001, r^2^ = 0.374). Regression line and 95% confidence interval shown. Purple circles= *Suessiales* spp, red circles= *Gymnodiniales* spp, green circles= *Prorocentrales* spp, orange circles= *Peridiniales* spp, blue circles= *Gonyaulacales* spp, yellow circles=*Dinophysiales* spp. Black triangles = data from the present study. **b** Relationship between log_10_ cell volume (µm^3^) and log_10_ rRNA copies cell^−1^ in dinoflagellates, from the present and previous studies (F = 0.26.59, df=106, *p* < 0.0001, r^2^ = 0.200). **c** Relationship between log_10_ cell volume (µm^3^) and log_10_ rRNA copies cell^−1^ in dinoflagellates, from published studies only (F = 16.4, df=58, *p* = 0.0002, r^2^ = 0.2204). [data from: 9, 10, 13, 19, 30, 38, 54, 55, 62, 63, 71, 74, 75, 76, 77, 78, 79, 81].

Estimates of CNV in other marine microbial eukaryotes suggested that variation of 1–3 orders of magnitude in rRNA, as was found in foraminifera, ciliates, haptophytes, fungi and diatoms, was considered very high [10–12], and was not always related to cell or genome size [10, 58]. In this study, rRNA CNV in one genus of a common harmful algae (*Alexandrium* spp) was found to span ~6 orders of magnitude. This should be considered the minimum variation, as we have studied comparatively few species of this genus. Previous estimates of genomic rRNA in *Alexandrium* spp indicated 10^2^–10^3^ copies cell^−1^ in *A. minutum*, [16]; from 10^4^–10^6^ copies cell^−1^ in *A. pacificum* [9], and *A. catenella* [17, 44, 45], consistent with our study. Very high rRNA GCN (10^6^) are also known from other Gonyaulacales spp [59, 60] measured using techniques including digital qPCR. Species of *Alexandrium* therefore span from amongst the lowest to the highest rRNA genomic copies cell^−1^ of dinoflagellates (Fig. 6a). In our study, most significantly, substantial intra-specific CNV was found among individuals collected from the same population (Fig. 4), of up to 2 orders of magnitude. Recently, ~1 order of magnitude CNV among 14 strains of the haptophyte *Emiliania huxleyi* from different global locations was reported [10]. Using single cell qPCR, two orders of magnitude difference in rRNA gene copies was found within a species of foraminifera [12]. Our results and these recent studies indicate that intraspecific rRNA CNV in marine microbial eukaryotes may be common, suggesting that rRNA gene copies cell^−1^ of multiple strains is required to determine reasonably representative GCN ranges. While CNV likely impacts analyses of all marine microbial eukaryotes, not addressing this will particularly impact dinoflagellate dominated assemblages, and assuming similarity in GCN in relation to the degree of phylogenetic relatedness appears to be ineffective.

### Sources of CNV: genome size variation, time in culture, and phylogenetic divergence

Differences in GCN can be the result of genome scale processes including polyploidy, as well as processes impacting part of the genome such as aneuploidy, chromosome duplications, errors during homologous recombination and retroposition [27, 31, 32]. Mutations and selection while held in laboratory culture could result in CNV, potentially with functional implications, as has been reported in bacteria and fungi, and specific processes may particularly impact rRNA loci [3, 61]. In rRNA genes, the presence of tandem repeats that are highly transcribed, and the difficulty of replicating repetitive sequences, make rDNA inherently less stable than other genes and susceptible to CNV [47]. Over cycles of cell division, rRNA GCN in yeast and bacteria in culture showed a pattern of reduction in fungi via recombination-mediated loss [62–64]. As well as these inherent genetic factors, environmental factors can lead to induced CNV in rRNA genes in some microbes [58, 61, 64, 65]. In the ciliates *Entodinium, Epidinium* and *Ophryoscolex*, GCN of rRNA changes in response to nutrient availability in cultures [65]. Higher rRNA GCN has ecological implications, providing protection against mutagens [62, 63] and impacting growth and competitive ability [58, 65].

We examined CNV in relation to generations in culture and geographic regions of isolation. Our strains spanned periods in culture of several months to ~30 years, yet no significant impact was found on either variability of genome size or rRNA CNV (Fig. 2, Supplementary Fig. 1), Intraspecific genetic diversity in rRNA gene copies in *A. pacificum* isolates from sites distant to one another was greater in natural populations than the amount induced by up to 30 years of laboratory selection (Fig. 4). The source of such CNV may be retroposition, as it appears common in dinoflagellates [21, 27, 31], and was found to be the source of multi-copies of *pcna* in dinoflagellates [66], as sequences had the remains of dinoflagellate specific spliced leader sequences, which are added to mRNA, evidence of reverse transcription into the genome. In Symbiodiniaceae, chromosomes may be enriched in relation to specific biological processes, suggesting that chromosomes may be duplicated or lost as required, creating CNV [28–30]. Whether through a process specific to rRNA, retroposition and/or chromosome duplications, CNV appears to occur over long evolutionary time scales based on our data, as dinoflagellate mutation rates on a decadal time scale were not a significant factor in the high CNV.

It has been calculated that <0.1% of species for which genome size data are available have genomes larger than 100 Gb [24], highlighting the exceptionally large genomes of dinoflagellates [21, 22, 24, 28–30]. In eukaryotes, there is no correlation between genome size and coding sequences in genomes larger than 0.01 Gb, known as the C-value enigma [21, 26, 67]. *Alexandrium* species were in the middle to upper half of reported dinoflagellate genome sizes (Fig. 1, Supplementary Fig. 2B), indicating expansion during evolution. Cell cycle synchronised genome sizes of *A. pacificum* varied ~ 3 fold (Fig. 1), greater than the already large range in previous reports of strains of this species (60–104) pg cell^−1^ [9, 22, 40, 68, 69]. Specialised ‘ribosomal chromosomes’ were found in the former *Alexandrium tamarense* species complex, including *A. pacificum* but not in *A. minutum*, [68], which in our study and previous studies showed much more consistent genome sizes among isolates (22–29 pg cell^−1^ in previous studies, as in this study [39, 40]). A significant relationship between genome size and rRNA gene copies^−1^ cell within *A. pacificum* (Fig. 6a) may indicate that rRNA chromosome duplication contributes to genome size expansion in this species [68], but appears unimportant within the other *Alexandrium* species examined.

Previous studies have measured dinoflagellate genomes using a variety of methods: flow cytometry, staining with propidium iodide or other DNA dyes, and using cells of chicken or a similarly sized animal genome as a standard, as well as estimates from genome sequencing (i.e. [22, 28–30, 39, 66, 67, 70], Supplementary Table 3). Genome size measurements based on flow cytometry using a standard of an animal genome, which is smaller and differs structurally from a dinoflagellate genome, may lead to larger genome size estimates as compared to estimates of sequenced dinoflagellate genomes (Supplementary Table 3). However, the pattern was not consistent, and some similar estimates were determined regardless of the method (Supplementary Table 3). Dinoflagellates have unusually large genomes, and therefore standards of the appropriate size are generally not available. We ensured that the standard curve used in this measurement achieved good linearity to minimise any potential error (Supplementary Materials).

### Implications for molecular quantification based on rRNA gene barcodes

Given the ubiquity of microbial eukaryotic ecological analyses routinely targeting rRNA genes for qPCR and meta-barcoding, it can appear as though such approaches are very well characterised [9, 10, 13, 17, 20, 56, 59, 71]. Considerable and justifiable attention has focused on sources of bias that confound universal rRNA gene markers, particularly PCR primer bias, sequencing bias and statistical bias [3, 72, 73]. Primers have been developed that address primer bias [72, 73], statistical approaches have addressed other biases, however less attention has been shown to GCN bias until recently [3, 74–76]. CNV is of particular concern for qPCR-based assays that are increasingly applied for the detection of HAB species such as *Alexandrium* spp [9, 16, 17, 44, 45, 56, 59, 60]. Previous estimates of cell abundance using rRNA-targeted qPCR of HAB species indicate both over and under-estimated abundances of up to 500% [45, 60, 77]. Despite the high variability in rRNA GCN within a population and species (Fig. 4), it is possible to calibrate a qPCR result against another type of environmental cell count via detailed light microscopy or fluorescently labelled flow cytometry[44, 45, 60]. A sampling design that integrated CNV of multiple populations co-occurring in a region could determine a local rRNA GCN as a calibration proxy. Because mutation rates of rRNA GCN were low over decades (Fig. 2), if a HAB species was seeded via local cyst beds, it may not be necessary to recalibrate with every sample, as doing so would render the simplicity, speed and scalability of qPCR-based quantification obsolete.

Community profiling using molecular barcoding presents a potentially more difficult challenge than qPCR in accommodating CNV, due to unknown variability between taxa and the lack of information on GCN. GCN correction factors are an option for addressing this, and can be incorporated into barcoding analyses [11, 74–76]. However, GCN data is very limited and patchily distributed. Current correction approaches rely on prediction using phylogenetically related taxa [65], however GCN of bacterial and fungal rRNA is only moderately phylogenetically conserved [58, 65, 74] resulting in increasingly inaccurate GCN predictions for diverging lineages [65]. The first study attempting to apply a GCN correction factor for marine microbial rRNA genes used a single mean GCN cell^−1^ for each class, from a database of ~60 species, of 4919 copies cell^−1^ for dinoflagellates, 166 copies cell^−1^ for diatoms and 71,710 copies cell^−1^ for ciliates [77]. While this is an improvement over using GCN as a direct proxy of ASV abundance, the level of variability found in our study shows that such an approach is limited. Genome sizes, potentially a proxy for GCN [Fig. 6a], are variable and unknown for most dinoflagellates, so this approach also appears to be unfeasible. [65] consider bioinformatic-based solutions to the CNV of rRNA genes is not realistic for microbial eukaryotes, and based on our current data, we agree with that position.

Meta-barcoding of microbial eukaryotes has been referred to as semi-quantitative, as it has been suggested that the relative abundance of taxa may be proportionately representative of the community [20]. However, analyses of bias in metabarcoding of dinoflagellate mock communities using multiple primers targeted to rRNA genes, or comparisons with microscopy counts, found ASV abundances were highly skewed [78, 79]. It has been argued that a lack of correlation between cell number and rRNA genes is unimportant, as rRNA gene copies cell^−1^ is correlated with cell volume [13, 19, 20, 69] in marine eukaryotes, providing a stronger indication of ecosystem function than cell abundance. However, we found only a weak relationship of rRNA gene copies cell^−1^ with cell volume in dinoflagellates, explaining only 20% of the variance (Fig. 6b, F = 0.26.59, df=106, *p* < 0.0001, r^2^ = 0.200). This was independent of our present study, as, even when we analysed published rRNA gene copy, cell volume and genome size data [9, 10, 16, 19, 22, 40, 59, 60, 67–70, 80–85] we found 22% of variance explained (Fig. 6c, F = 16.4, df=58, *p* = 0.0002, r^2^ = 0.2204). When analysing only rRNA copies cell^−1^ in relation to cell volume for Gonyaulacales spp, the order including many of the most important HAB taxa, we found no significant relationship (Fig. 6c, Supplementary Fig. 2, F = 3.26, df=85, p=ns, r^2^ = 0.037). This may be indicative of high evolutionary rates of rRNA genes in this order, noted in relation to long branch lengths in rRNA gene-based phylogenies [86]. Dinoflagellates with the smallest genomes, such as *Suessiales* spp, appear to show a more straightforward relationship between genome size, cell volume and GCN (Fig. 6a–c), but polyploidy and chromosome duplication are still known from these taxa [85].

Given that ecosystem function is generally of primary interest in microbial ecological research, we suggest metabarcoding be considered a diversity presence/absence measure, and functional genes be quantified using metagenomics or gene specific assays in relation to traits of interest. Our data indicate that comparably fewer copies of the gene related to PST synthesis, *sxtA4*, were present in PST-producing strains (2–10^2^ copies cell^−1^) in *Alexandrium*, with a smaller range of CNV (Fig. 3c), consistent with studies of *sxtA4* in *A. minutum* (1.5–46 copies cell^−1^; [39, 40]) and *A. pacificum* (34–200 copies cell^−^^1^; [37, 40, 44]). Both the reduced CNV, combined with the significant correlation of *sxtA4* GCN with PSTs cell^−1^ indicate that *sxtA4* is an informative target for the potential for PST production in situ. We found a variety of PST analogues produced by *Alexandrium* species, with some species such as *A.catenella* more consistent in analogues produced than others (Fig. 5). *sxtA* is involved in the synthesis of the parent compound, saxitoxin, [36, 37] and tailoring enzymes in the *sxt* cluster appear to be responsible for analogues, suggesting that in future these genes could be quantified. An advantage of the *sxtA* detection is that food web dynamics can be investigated, for example, quantifying *sxtA* uptake in invertebrates or protists [87]. Examples of similar functional genes that have been or could be detected *in situ* are cell cycle related genes such as *pcna* involved in proliferation and growth of HAB species [66], transporters, receptors, genes involved in N and P uptake and other metabolic functions [13, 21, 29, 30, 85]. Given the potential for such genes to show a dosage response in dinoflagellates, this could indicate a promising approach to community ecology in dinoflagellate dominated ecosystems.

## Conclusion

We have shown rRNA gene CNV of up to 6 orders of magnitude in dinoflagellates at the class, genus and species levels, and its relationship to genome size, but not generations in laboratory culture, and very weakly with cell volume. Reported significant correlations between cell volume and rRNA genomic copies in marine microbial eukaryotes were likely driven by taxa with more straightforward genome organisation, rather than dinoflagellates, ciliates and foraminifera, which taken together can constitute the majority of 18 S rRNA signal. We show a significant dosage effect of a functional gene related to a common HAB toxin, and suggest that such low copy functional genes are more stable and informative targets for eukaryotic microbial ecological profiling, using function-based methods such as metagenomics and specific trait-based molecular assays.

## Supplementary information

Supplementary Figures & Tables

Supplementary Table 1

## Data Availability

Data will be made available for all non-commercial purposes.

## References

[1] Hardigan MA, Crisovan E, Hamilton JP, Kim J, Laimbeer P, Leisner CP (2016). Genome reduction uncovers a large dispensable genome and adaptive role for copy nmber vriation in aexually popagated Solanum tuberosum. Plant Cell.

[2] Perry GH, Dominy NJ, Claw KG, Lee AS, Fiegler H, Redon R (2007). Diet and the evolution of human amylase gene copy number variation. Nat Genet.

[3] Soppa J (2017). Polyploidy and community structure. Nat Microbiol.

[4] Conrad DF, Pinto D, Redon R, Feuk L, Gokcumen O, Zhang Y (2010). Origins and functional impact of copy number variation in the human genome. Nature.

[5] Zerulla K, Soppa J (2014). Polyploidy in haloarchaea: advantages for growth and survival. Front Microbiol.

[6] Iantorno SA, Durrant C, Khan A, Sanders MJ, Beverley SM, Warren WC (2021). Gene expression in leishmania is regulated predominantly by gene dosage. mBio.

[7] Gillard GB, Gronvold L, Rosaeg LL, Holen MM, Monsen O, Koop BF (2021). Comparative regulomics supports pervasive selection on gene dosage following whole genome duplication. Genome Biol.

[8] Keeling PJ, Campo JD (2017). Marine protists are not just big bacteria. Curr Biol.

[9] Galluzzi L, Bertozzini E, Penna A, Perini F, Garcés E, Magnani M (2010). Analysis of rRNA gene content in the Mediterranean dinoflagellate Alexandrium catenella and Alexandrium taylori: implications for the quantitative real-time PCR-based monitoring methods. J Appl Phycol.

[10] Gong W, Marchetti A (2019). Estimation of 18S gene copy number in marine eukaryotic plankton using a next-generation sequencing approach. Front Marine Sci.

[11] Wang C, Zhang T, Wang Y, Katz LA, Gao F, Song W (2017). Disentangling sources of variation in SSU rDNA sequences from single cell analyses of ciliates: impact of copy number variation and experimental error. Proc Biol Sci.

[12] Milivojevic T, Rahman SN, Raposo D, Siccha M, Kucera M, Morard R (2021). High variability in SSU rDNA gene copy number among planktonic foraminifera revealed by single-cell qPCR. ISME Commun.

[13] de Vargas C, Audic S, Henry N, Decelle J, Mahé F, Logares R (2015). Ocean plankton. Eukaryotic plankton diversity in the sunlit ocean. Science.

[14] Thornhill DJ, Lajeunesse TC, Santos SR (2007). Measuring rDNA diversity in eukaryotic microbial systems: how intragenomic variation, pseudogenes, and PCR artifacts confound biodiversity estimates. Mol Ecol.

[15] Egge E, Bittner L, Andersen T, Audic S, de Vargas C, Edvardsen B (2013). 454 pyrosequencing to describe microbial eukaryotic community composition, diversity and relative abundance: a test for marine haptophytes. PLoS One.

[16] Galluzzi L, Penna A, Bertozzini E, Vila M, Garces E, Magnani M (2004). Development of a real-time PCR assay for rapid detection and quantification of Alexandrium minutum (a Dinoflagellate). Appl Environ Microbiol.

[17] Erdner DL, Percy L, Keafer B, Lewis J, Anderson DM (2010). A quantitative real-time PCR assay for the identification and enumeration of Alexandrium cysts in marine sediments. Deep Sea Res Part 2 Top Stud Oceanogr.

[18] Prokopowich CD, Gregory TR, Crease TJ (2003). The correlation between rDNA copy number and genome size in eukaryotes. Genome.

[19] Godhe A, Asplund ME, Harnstrom K, Saravanan V, Tyagi A, Karunasagar I (2008). Quantification of diatom and dinoflagellate biomasses in coastal marine seawater samples by real-time PCR. Appl Environ Microbiol.

[20] Le Bescot N, Mahé F, Audic S, Dimier C, Garet MJ, Poulain J (2016). Global patterns of pelagic dinoflagellate diversity across protist size classes unveiled by metabarcoding. Environ Microbiol.

[21] Lin S (2011). Genomic understanding of dinoflagellates. Res Microbiol.

[22] LaJeunesse TC, Lambert G, Andersen RA, Coffroth MA, Galbraith DW (2005). Symbiodinium (Pyrrhophyta) genome sizes (DNA Content) are smallest among dinoflagellates. J Phycol.

[23] Hackett JD, Scheetz TE, Yoon HS, Soares MB, Bonaldo MF, Casavant TL (2005). Insights into a dinoflagellate genome through expressed sequence tag analysis. BMC Genomics.

[24] Hidalgo O, Pellicer J, Christenhusz M, Schneider H, Leitch AR, Leitch IJ (2017). Is There an Upper Limit to Genome Size?. Trends Plant Sci.

[25] Pellicer J, Hidalgo O, Dodsworth S, Leitch IJ (2018). Genome size diversity and its impact on the evolution of land plants. Genes (Basel).

[26] Murray SA, Suggett DJ, Doblin MA, Kohli GS, Seymour JR, Fabris M (2016). Unravelling the functional genetics of dinoflagellates: a review of approaches and opportunities. Perspect Phycol.

[27] Bachvaroff TR, Place AR (2008). From stop to start: tandem gene arrangement, copy number and trans-splicing sites in the dinoflagellate Amphidinium carterae. PLoS One.

[28] Liu H, Stephens TG, Gonzalez-Pech RA, Beltran VH, Lapeyre B, Bongaerts P (2018). Symbiodinium genomes reveal adaptive evolution of functions related to coral-dinoflagellate symbiosis. Commun Biol.

[29] Lin S, Cheng S, Song B, Zhong X, Lin X, Li W (2015). The Symbiodinium kawagutii genome illuminates dinoflagellate gene expression and coral symbiosis. Science.

[30] Stephens TG, Gonzalez-Pech RA, Cheng Y, Mohamed AR, Burt DW, Bhattacharya D (2020). Genomes of the dinoflagellate Polarella glacialis encode tandemly repeated single-exon genes with adaptive functions. BMC Biol.

[31] Slamovits CH, Keeling PJ (2008). Widespread recycling of processed cDNAs in dinoflagellates. Curr Biol.

[32] Song B, Chen S, Chen W (2018). Dinoflagellates, a unique lineage for retrogene research. Front Microbiol.

[33] Goetz EJ, Greco M, Rappaport HB, Weiner AKM, Walker LM, Bowser S (2022). Foraminifera as a model of the extensive variability in genome dynamics among eukaryotes. Bioessays.

[34] Kouakou CRC, Poder TG (2019). Economic impact of harmful algal blooms on human health: a systematic review. J Water Health.

[35] Ryderheim F, Selander E, Kiorboe T (2021). Predator-induced defence in a dinoflagellate generates benefits without direct costs. ISME J.

[36] Hackett JD, Wisecaver JH, Brosnahan ML, Kulis DM, Anderson DM, Bhattacharya D (2013). Evolution of saxitoxin synthesis in cyanobacteria and dinoflagellates. Mol Biol Evol.

[37] Stuken A, Orr RJ, Kellmann R, Murray SA, Neilan BA, Jakobsen KS (2011). Discovery of nuclear-encoded genes for the neurotoxin saxitoxin in dinoflagellates. PLoS One.

[38] Orr RJ, Stuken A, Murray SA, Jakobsen KS (2013). Evolution and distribution of saxitoxin biosynthesis in dinoflagellates. Mar Drugs.

[39] Stuken A, Riobo P, Franco J, Jakobsen KS, Guillou L, Figueroa RI (2015). Paralytic shellfish toxin content is related to genomic sxtA4 copy number in Alexandrium minutum strains. Front Microbiol.

[40] Geffroy S, Lechat MM, Le Gac M, Rovillon GA, Marie D, Bigeard E (2021). From the sxtA4 gene to saxitoxin production: what controls the variability among alexandrium minutum and alexandrium pacificum strains?. Front Microbiol.

[41] Savela H, Harju K, Spoof L, Lindehoff E, Meriluoto J, Vehniainen M (2016). Quantity of the dinoflagellate sxtA4 gene and cell density correlates with paralytic shellfish toxin production in Alexandrium ostenfeldii blooms. Harmful Algae.

[42] Perini F, Galluzzi L, Dell’Aversano C, Iacovo ED, Tartaglione L, Ricci F (2014). SxtA and sxtG gene expression and toxin production in the Mediterranean Alexandrium minutum (Dinophyceae). Mar Drugs.

[43] Wiese M, Murray SA, Alvin A, Neilan BA (2014). Gene expression and molecular evolution of sxtA4 in a saxitoxin producing dinoflagellate Alexandrium catenella. Toxicon.

[44] Murray SA, Wiese M, Stuken A, Brett S, Kellmann R, Hallegraeff G (2011). sxtA-based quantitative molecular assay to identify saxitoxin-producing harmful algal blooms in marine waters. Appl Environ Microbiol.

[45] Murray SA, Ruvindy R, Kohli GS, Anderson DM, Brosnahan ML (2019). Evaluation of sxtA and rDNA qPCR assays through monitoring of an inshore bloom of Alexandrium catenella Group 1. Sci Rep.

[46] Murray SA, Diwan R, Orr RJ, Kohli GS, John U (2015). Gene duplication, loss and selection in the evolution of saxitoxin biosynthesis in alveolates. Mol Phylogenet Evol.

[47] Salim D, Gerton JL (2019). Ribosomal DNA instability and genome adaptability. Chromosome Res.

[48] Keller MD, Selvin RC, Claus W, Guillard RRL (1987). Media for the culture of oceanic ultraphytoplankton. J Phycol.

[49] Scholin CA, Herzog M, Sogin M, Anderson DM (1994). Identification of group- and strain-specific genetic markers for globally distributed Alexandrium (Dinophyceae).II. Sequence analysis of a fragment of the LSU rRNA gene. J Phycol.

[50] Nunn GB, Theisen BF, Christensen B, Arctander P (1996). Simplicity-correlated size growth of the nuclear 28S ribosomal RNA D3 expansion segment in the crustacean order Isopoda. J Mol Evol.

[51] Doblin MA, Blackburn SI, Hallegraeff GM (1999). Growth and biomass stimulation of the toxic dinoflagellate Gymnodinium catenatum (Graham) by dissolved organic substances. J Exp Mar Bio Ecol.

[52] Taroncher-Oldenburg G, Kulis DM, Anderson DM (1997). Toxin variability during the cell cycle of the dinoflagellate Alexandrium fundyense. Limnol Oceanogr.

[53] Figueroa RI, Garcés E, Bravo I (2010). The use of flow cytometry for species identification and life-cycle studies in dinoflagellates. Deep Sea Res Part 2 Top Stud Oceanogr.

[54] Vindelov LL, Christensen IJ, Nissen NI (1983). Standardization of high-resolution flow cytometric DNA analysis by the simultaneous use of chicken and trout red blood cells as internal reference standards. Cytometry.

[55] Doležel J, Bartoš J, Voglmayr H, Greilhuber J (2003). Nuclear DNA content and genome size of trout and human. Cytometry Part A.

[56] Ruvindy R, Bolch CJ, MacKenzie L, Smith KF, Murray SA (2018). qPCR assays for the detection and quantification of multiple paralytic shellfish toxin-producing species of Alexandrium. Front Microbiol.

[57] Harwood DT, Boundy M, Selwood AI, van Ginkel R (2013). Refinement and implementation of the Lawrence method (AOAC 2005.06) in a commercial laboratory: assay performance during an Alexandrium catenella bloom event. Harmful Algae.

[58] Lofgren LA, Uehling JK, Branco S, Bruns TD, Martin F, Kennedy PG (2019). Genome-based estimates of fungal rDNA copy number variation across phylogenetic scales and ecological lifestyles. Mol Ecol.

[59] Yarimizu K, Sildever S, Hamamoto Y, Tazawa S, Oikawa H, Yamaguchi H (2021). Development of an absolute quantification method for ribosomal RNA gene copy numbers per eukaryotic single cell by digital PCR. Harmful Algae.

[60] Nishimura T, Hariganeya N, Tawong W, Sakanari H, Yamaguchi H, Adachi M (2016). Quantitative PCR assay for detection and enumeration of ciguatera-causing dinoflagellate Gambierdiscus spp. (Gonyaulacales) in coastal areas of Japan. Harmful Algae.

[61] Nelson JO, Watase GJ, Warsinger-Pepe N, Yamashita YM (2019). Mechanisms of rDNA copy number maintenance. Trends Genet.

[62] Ide S, Miyazaki T, Maki H, Kobayashi T (2010). Abundance of ribosomal RNA gene copies maintains genome integrity. Science.

[63] Kobayashi T (2011). Regulation of ribosomal RNA gene copy number and its role in modulating genome integrity and evolutionary adaptability in yeast. Cell Mol Life Sci.

[64] Nemergut DR, Knelman JE, Ferrenberg S, Bilinski T, Melbourne B, Jiang L (2016). Decreases in average bacterial community rRNA operon copy number during succession. ISME J.

[65] Lavrinienko A, Jernfors T, Koskimäki JJ, Pirttilä AM, Watts PC (2021). Does intraspecific variation in rDNA copy number affect analysis of microbial communities?. Trends in Microbiology.

[66] Hou Y, Ji N, Zhang H, Shi X, Han H, Lin S (2019). Genome size-dependent pcna gene copy number in dinoflagellates and molecular evidence of retroposition as a major evolutionary mechanism. J Phycol.

[67] Hou Y, Lin S (2009). Distinct gene number-genome size relationships for eukaryotes and non-eukaryotes: gene content estimation for dinoflagellate genomes. PLoS One.

[68] Figueroa RI, Cuadrado A, Stüken A, Rodríguez F, Fraga S (2014). Ribosomal DNA organization patterns within the dinoflagellate genus Alexandrium as revealed by FISH: life cycle and evolutionary implications. Protist.

[69] Liu Y, Hu Z, Deng Y, Shang L, Gobler CJ, Tang YZ (2021). Dependence of genome size and copy number of rRNA gene on cell volume in dinoflagellates. Harmful Algae.

[70] Kohli GS, John U, Figueroa RI, Rhodes LL, Harwood DT, Groth M (2015). Polyketide synthesis genes associated with toxin production in two species of Gambierdiscus (Dinophyceae). BMC Genomics.

[71] Ott BM, Litaker RW, Holland WC, Delwiche CF (2022). Using RDNA sequences to define dinoflagellate species. PLOS ONE.

[72] McNichol J, Berube PM, Biller SJ, Fuhrman J A Evaluating and Improving Small Subunit rRNA PCR Primer Coverage for Bacteria, Archaea, and Eukaryotes Using Metagenomes from Global Ocean Surveys. mSystems 2021; 6: 10.1128/msystems.00565-21. 10.1128/mSystems.00565-21 PMC8269242 34060911

[73] Parada AE, Needham DM, Fuhrman JA (2016). Every base matters: assessing small subunit rRNA primers for marine microbiomes with mock communities, time series and global field samples. Environ Microbiol.

[74] Gonzalez-de-Salceda L, Garcia-Pichel F (2021). The allometry of cellular DNA and ribosomal gene content among microbes and its use for the assessment of microbiome community structure. Microbiome.

[75] Louca S, Doebeli M, Parfrey LW (2018). Correcting for 16S rRNA gene copy numbers in microbiome surveys remains an unsolved problem. Microbiome.

[76] Stoddard SF, Smith BJ, Hein R, Roller BRK, Schmidt TM (2015). rrnDB: improved tools for interpreting rRNA gene abundance in bacteria and archaea and a new foundation for future development. Nucleic Acids Res.

[77] Godhe A, Cusack C, Pedersen J, Andersen P, Anderson DM, Bresnan E (2007). Intercalibration of classical and molecular techniques for identification of Alexandrium fundyense (Dinophyceae) and estimation of cell densities. Harmful Algae.

[78] Smith KF, Kohli GS, Murray SA, Rhodes LL (2017). Assessment of the metabarcoding approach for community analysis of benthic-epiphytic dinoflagellates using mock communities. N Z J Marine Freshwater Res.

[79] Santi I, Kasapidis P, Karakassis I, Pitta P (2021). A comparison of DNA metabarcoding and microscopy methodologies for the study of aquatic microbial eukaryotes. Diversity.

[80] Martin JL, Santi I, Pitta P, John U, Gypens N (2022). Towards quantitative metabarcoding of eukaryotic plankton: an approach to improve 18S rRNA gene copy number bias. MBMG.

[81] Kretzschmar AL, Verma A, Kohli G, Murray S (2019). Development of a quantitative PCR assay for the detection and enumeration of a potentially ciguatoxin-producing dinoflagellate, Gambierdiscus lapillus (Gonyaulacales, Dinophyceae). PLOS ONE.

[82] Menden-Deuer S, Lessard EJ (2000). Carbon to volume relationships for dinoflagellates, diatoms, and other protist plankton. Limnol Oceanogr.

[83] Zhu F, Massana R, Not F, Marie D, Vaulot D (2005). Mapping of picoeucaryotes in marine ecosystems with quantitative PCR of the 18S rRNA gene. FEMS Microbiol Ecol.

[84] Vandersea MW, Kibler SR, Holland WC, Tester PA, Schultz TF, Faust MA (2012). Development of semi-quantitative PCR assays for the detection and enumeration of *Gambierdiscus* species (Gonyaulacales, Dinophyceae). J Phycol.

[85] Nand A, Zhan Y, Salazar OR, Aranda M, Voolstra CR, Dekker J (2021). Genetic and spatial organization of the unusual chromosomes of the dinoflagellate Symbiodinium microadriaticum. Nature Genetics.

[86] Kretzschmar AL, Verma A, Murray S, Kahlke T, Fourment M, Darling AE. Trial by phylogenetics—Evaluating the Multi-Species Coalescent for phylogenetic inference on taxa with high levels of paralogy (Gonyaulacales, Dinophyceae). bioRxiv. 10.1101/683383.

[87] Farrell H, O’Connor WA, Seebacher F, Harwood TD, Murray S (2016). Molecular detection of the SxtA gene from saxitoxin-producing Alexandrium minutum in commercial oysters. J Shellfish Res.

