# Supplementary material for: Genomic copy number variability at the genus, species and population levels impacts in situ ecological analyses of dinoflagellates and harmful algal blooms
Source: ISME Commun. 2023 Jul 8;3:70. doi: 10.1038/s43705-023-00274-0 (PMC10329664; doi:10.1038/s43705-023-00274-0)
Supplement: Supplementary file 1 — Supplementary Figures & Tables [file 43705_2023_274_MOESM1_ESM.docx]

**Supplementary Figures & Tables**


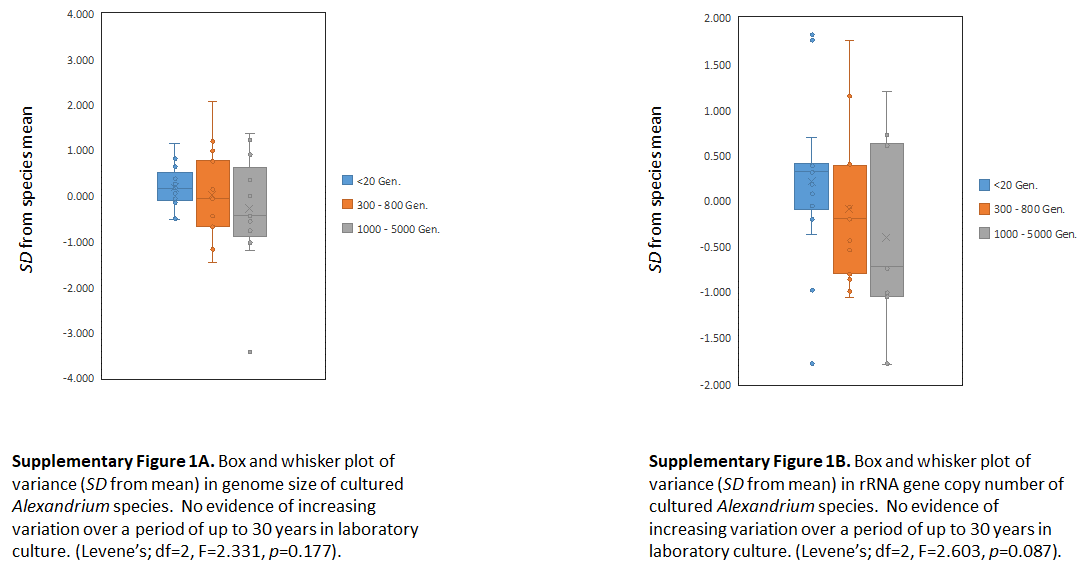


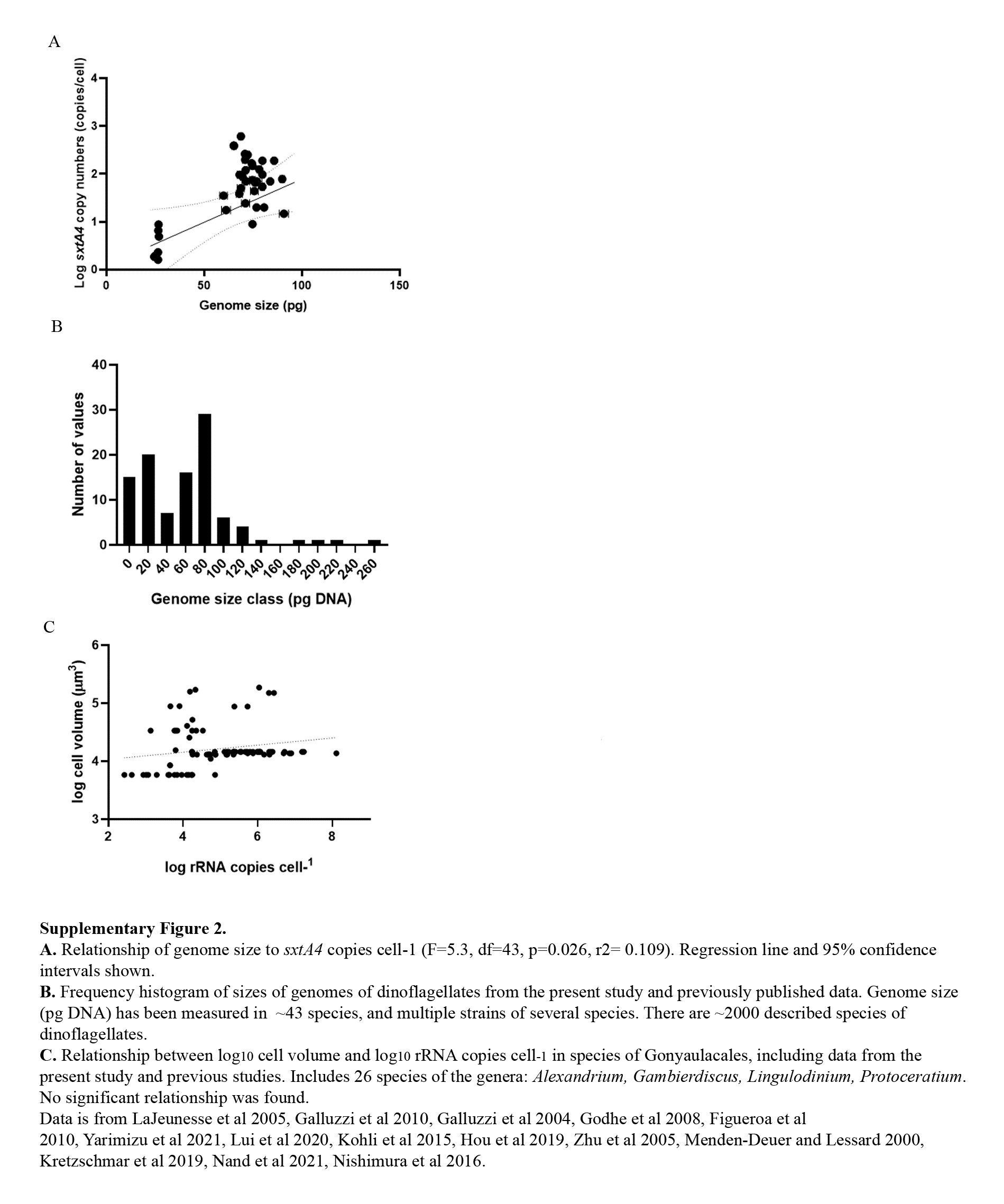


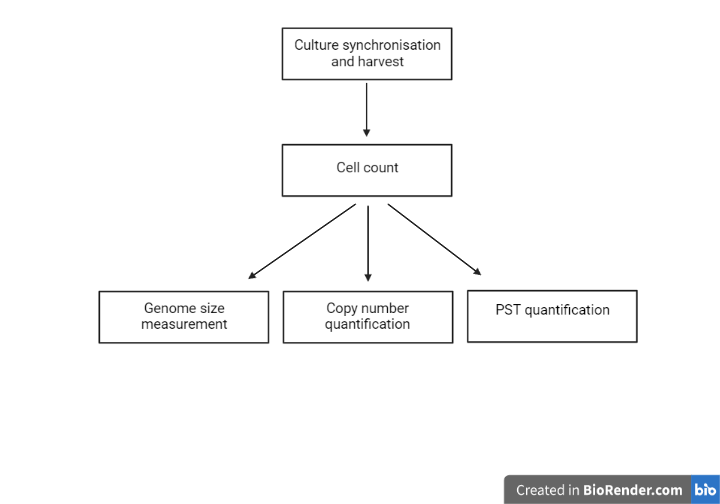


Supplementary Figure 3. Workflow chart for this study.


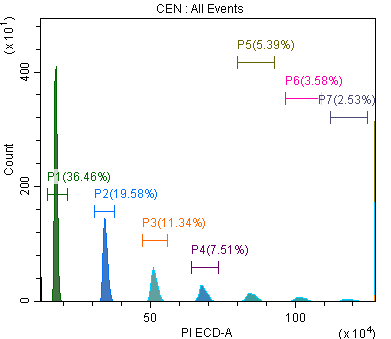


Supplementary Figure 4. Histogram showing the fluorescence peaks each with the percentage of events used in the flow cytometric analyses of genome size.


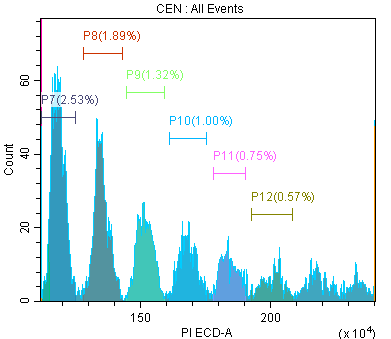


Supplementary Figure 5. Histogram in zoom showing the rest of the fluorescence peaks used in the flow cytometric analyses of genome size.

Supplementary Table 2. Fluorescence Intensity Values Generated by the CEN Standard in the flow cytometric analyses of genome size.

|  | **Genome Size pg/nuclei)** | **Mean PI ECD-A** | | | **Average** |
| --- | --- | --- | --- | --- | --- |
|  |  | **Std1** | **Std2** | **Std3** |  |
| P1 | 3 | 191158 | 190220.4 | 188980.2 | 190119.5333 |
| P2 | 6 | 378821.5 | 309495.4 | 375780.7 | 354699.2 |
| P3 | 9 | 565447.1 | 564314.2 | 561272.8 | 563678.0333 |
| P4 | 12 | 751746.9 | 751343.9 | 746292.3 | 749794.3667 |
| P5 | 15 | 936358 | 935316.9 | 929405.9 | 933693.6 |
| P6 | 18 | 1120202.5 | 1118320.3 | 1112065.3 | 1116862.7 |
| P7 | 21 | 1307795.4 | 1304934.9 | 1297231.6 | 1303320.633 |
| P8 | 24 | 1487987.1 | 1490052 | 1477462.8 | 1485167.3 |
| P9 | 27 | 1674091.6 | 1665255 | 1663889.9 | 1667745.5 |
| P10 | 30 | 1837889.8 | 1840841.9 | 1824134.3 | 1834288.667 |
| P11 | 33 | 1996170.1 | 2020630.6 | 2023269.5 | 2013356.733 |
| P12 | 36 | 2202388 | 2199779.3 | 2196122 | 2199429.767 |


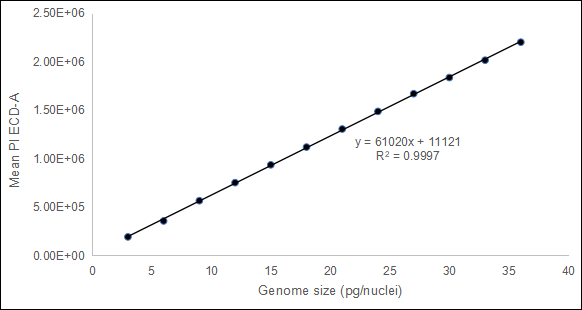


Supplementary Figure 6. Chicken blood cell standard curve for genome size as measured using flow cytometry.


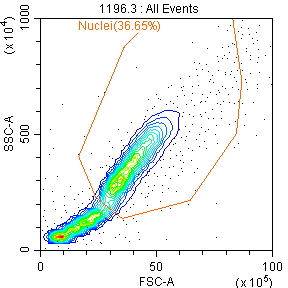

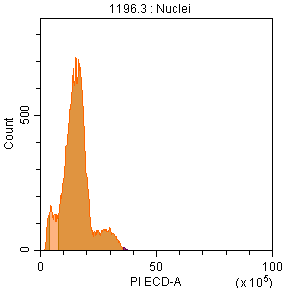


Supplementary Figure 7. Flow cytometry gating. This shows the gating area established to separate the nucleus and debris.


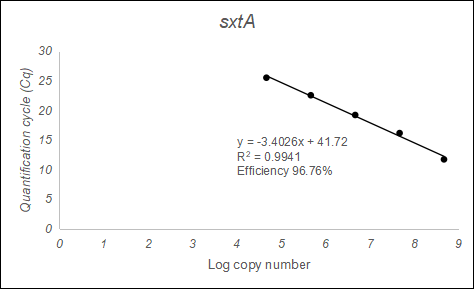


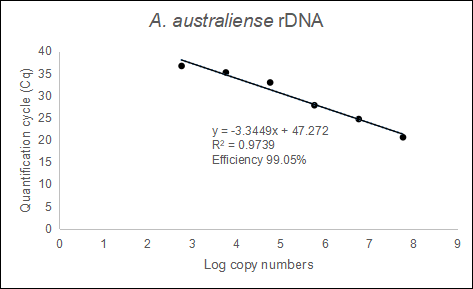


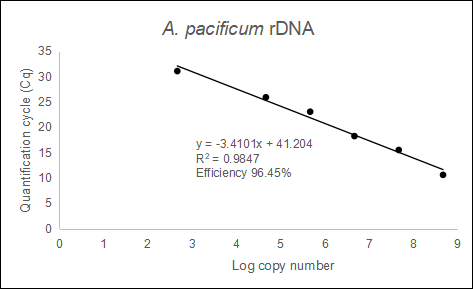


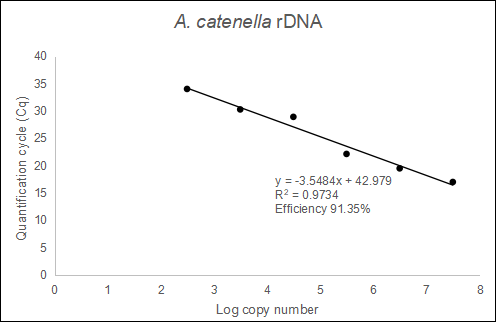


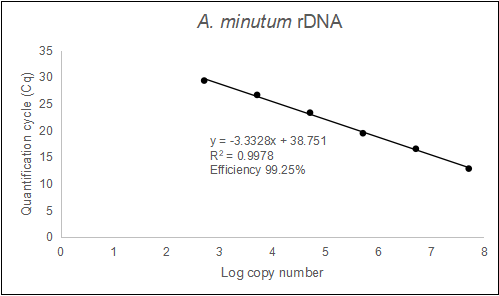


Supplementary Figure 8. qPCR standard curves. This shows assays had efficiencies between the acceptable ranges of 90-100%.

Supplementary Table 3. Genome size estimation comparison between La Jeunesse et al 2005 (using flow cytometry and a chicken blood cell standard) and other methods.

| **Genus/species** | **Genome size estimation (flow cytometry, chicken blood cell standards), La Jeunesse et al 2005.** | **Genome size estimation (sequencing, other methods)** |
| --- | --- | --- |
| *Amphidinium carterae* | 5.7 Gb |  |
| *Polarella glacialis* | 6.7 Gb | 3.0 Gb diploid (Stephens et al 2020) |
| *Alexandrium tamarense* | 99.9 Gb |  |
| *Symbiodinium genus (Clade A)* | 2.1-3.3 Gb | 0.7 – 2.0 Gb (Gonzalez Pech et al 2021) |
| *Breviolum genus (Clade B)* | 1.4 -2.7 Gb | 1.8-2.0 (Saad et al 2020), 1.5 (Shoguchi et al 2013) |
| *Cladocopium genus (Clade C)* | 2.8-4.6 Gb | 3-5 Gb (Saad et al 2020), 1.3 Gb (Chen et al 2022) |
| *Durusdinium trenchii (D1a)* | 3.4-3.9 Gb | 3-4 Gb (Saad et al 2020), 1.1-1.6 Gb (Dougan et al 2022a) |
| *Effrenium voratum (Clade E)* | 3.2 Gb | 2.5-3.0 Gb (Saad et al 2020), 1.2-1.9 Gb (Shah et al. 2023) |
| *Fugacium genus (Clade F)* | 2.9 Gb | 2.5 Gb (Saad et al 2020), 1.2 (Lin et al. 2015; Liu et al 2018) |
| *Prorocentrum cordatum* | 6.6 Gb | 4.75 Gb (Dougan et al 2022b) |

References

González-Pech, R.A., Stephens, T.G., Chen, Y., Mohamed, A.R., Cheng, Y., Shah, S., Dougan, K.E., Fortuin, M.D., Lagorce, R., Burt, D.W. and Bhattacharya, D., 2021. Comparison of 15 dinoflagellate genomes reveals extensive sequence and structural divergence in family Symbiodiniaceae and genus *Symbiodinium*. BMC Biology, 19, pp.1-22.

Shoguchi, E., Shinzato, C., Kawashima, T., Gyoja, F., Mungpakdee, S., Koyanagi, R., Takeuchi, T., Hisata, K., Tanaka, M., Fujiwara, M. and Hamada, M., 2013. Draft assembly of the *Symbiodinium minutum* nuclear genome reveals dinoflagellate gene structure. Current biology, 23(15), pp.1399-1408.

Stephens, T.G., González-Pech, R.A., Cheng, Y., Mohamed, A.R., Burt, D.W., Bhattacharya, D., Ragan, M.A. and Chan, C.X., 2020. Genomes of the dinoflagellate *Polarella glacialis* encode tandemly repeated single-exon genes with adaptive functions. BMC Biology, 18(1), pp.1-21.

Liu, H., Stephens, T.G., González-Pech, R.A., Beltran, V.H., Lapeyre, B., Bongaerts, P., Cooke, I., Aranda, M., Bourne, D.G., Forêt, S. and Miller, D.J., 2018. *Symbiodinium* genomes reveal adaptive evolution of functions related to coral-dinoflagellate symbiosis. Communications biology, 1(1), p.95.

LaJeunesse, T.C., Lambert, G., Andersen, R.A., Coffroth, M.A. and Galbraith, D.W., 2005. *Symbiodinium* (pyrrhophyta) genome sizes (DNA content) are smallest among dinoflagellates 1. Journal of Phycology, 41(4), pp.880-886.

Saad, O.S., Lin, X., Ng, T.Y., Li, L., Ang, P. and Lin, S., 2020. Genome size, rDNA copy, and qPCR assays for Symbiodiniaceae. Frontiers in Microbiology, 11, p.847.

Supplementary Table 4. Copy number, Genome size, and PST measurement summary.

|  | **Species name** |  | |  | **Copy number variation (copies/cell)** | | |  |  |  |
| --- | --- | --- | --- | --- | --- | --- | --- | --- | --- | --- |
|  |  |  | |  |  | **rRNA** | ***sxtA*** | **genome size (pg/cell)** | **PST (ng/cell)** | |
|  | *Alexandrium* | *catenella* | |  | Max | 2073024.6 | 189.1 | 89.9 | 0.1261 |  |
|  |  |  | |  | Min | 18008.1 | 19.9 | 68.13 | 0.0144 |  |
|  |  |  | |  | Mean | 360247.1 | 80.4 | 79.12 | 0.0402 |  |
|  |  |  | |  |  |  |  |  |  |  |
|  | *Alexandrium* | *pacificum* | |  | Max | 17253028 | 609 | 130.9 | 0.0096 |  |
|  |  | |  |  | Min | 17083.6 | 7.8 | 42.35 | 0.0011 |  |
|  |  | |  |  | Mean | 12467068 | 138.4 | 72.55 | 0.0045 |  |
|  |  | |  |  |  |  |  |  |  |  |
|  | *Alexandrium* | | *minutum* |  | Max | 17596.9 | 8.8 | 30.65 | 0.2724 |  |
|  |  | |  |  | Min | 267.3 | 0 | 22.55 | 0 |  |
|  |  | |  |  | Mean | 11147.9 | 1.9 | 27.02 | 0.0242 |  |
|  |  | |  |  |  |  |  |  |  |  |
|  | *Alexandrium* | | *australiense* | | Max | 123653553 | 8.9 | 96.07 | 0.0008 |  |
|  |  | |  |  | Min | 1574075 | 0 | 74.73 | 0 |  |
|  |  | |  |  | Mean | 33949483 | 2.3 | 87.32 | 0.0004 |  |
|  |  | |  |  |  |  |  |  |  |  |

Supplementary Table 5. Sequences of Primers and Gene Fragments Used in Gene Copy Number Quantification.

| Species | Target gene | Primers | References | Gene fragment (gBlocks®) Sequence | Amplicon size |
| --- | --- | --- | --- | --- | --- |
|  | *sxtA* | 5'-ctg agc aag gcg ttc aat tc-3' | Murray et al. 2011 | cttcgggttg gactacgcgg agaacaacat catctacgcc gggcagctga gcaaggcgtt caattcgccc ggcggattcg tcagctgtgc gcgcgagacc gacgagaatt tcggcgttct gaacttggcc aagaactcga acacactcgt gttcacaggg ccgatctgta ctgccggcct gtcgagtgcg aagacgacct | 124 bp |
|  |  | 5'-tac aga tmg gcc ctg tga rc -3' |  |  |  |
| *A. pacificum* | 28S rDNA | 5’-tcc tca gtg aga ttg tag tg-3’ | Ruvindy et al. 2018 | aatgagtttg tatttgctaa acacaaagta aacagacttg atttcctcag tgagattgta gtgcttgctt aacaatgggt tttggctgca agtgcaataa ttcttgcttt gtgtgccagt ttttatgtgg acatttgatt acctttgcac atgaatggta attttcctgc ggggtgtgga ttgcatatgc atgtaatgat ttgcatgttt gttaaatgtg tctggtgtat ttgtttgtgt ccttgtcctt gaggttgctt tctcccttgg gcttacatgc | 204 bp |
|  |  | 5’-gac aag gac aca aac aaa tac-3’ |  |  |  |
| *A. catenella* | 28S rDNA | 5’-tga ttt gct tgg tgg gag tg-3’ | Ruvindy et al. 2018 | gagtttgtat ttgctgaaca caaagtaaac agacttgatt tgcttggtgg gagtgttgca cttgcttgac aagagctttg ggctgtgggc gtaatgattc tttctttgca tgccagtttc tatgtgtaca tctgattacc tttgcacatg aatgataagt ctcctgtggg gggtggattg catgtgcatg taatgatttg tgttttgata aatgtgtctg gtgtatgtgt gtgtgttcct gtgcttgggg atgcttcctt ccttggactt acaagccctg acacacacat gctggcaaaa | 229 bp |
|  |  | 5’-caa gga agg aag cat ccc c-3’ |  |  |  |
| *A. australiense* | 28S rDNA | 5’-cgg tgg gtg caa tga ttc-3’ | Ruvindy et al. 2018 | cttagtgaga ttgtagcact tgcttgacaa taggttttgg cggtgggtgc aatgattctt gctttgtatg ccagtttcta tgtggacatt tgattacctg tgcacttgaa tggtaatttt cctgcggggg gtggattgca tatgcatgta attatttgca | 85 bp |
|  |  | 5’-gca gga aaa tta cca ttc aag t-3’ |  |  |  |
| *A. minutum* | 18S rDNA | 5'-aca tgg ata act gtg gta att cta tag cta a-3' | Toebe et al. 2013 | gctcattaaa acagttataa tgcacttgat gatcgattgc ttacatggat aactgtggta attctatagc taatacatgc atccaaacct gacttctggg aagggttgtg gtcattagtt acagaaccaa cccaggctct gcttggaatt ttggtgattc atgatgactg aatgaattac | 89 bp |
|  |  | 5'-gtt ggt tct gta act aat gac cac aac-3' |  |  |  |
