# Supplementary material for: Genomic copy number variability at the genus, species and population levels impacts in situ ecological analyses of dinoflagellates and harmful algal blooms
Source: ISME Commun. 2023 Jul 8;3:70. doi: 10.1038/s43705-023-00274-0 (PMC10329664; doi:10.1038/s43705-023-00274-0)
Supplement: Supplementary file 2 — Supplementary Table 1 [file 43705_2023_274_MOESM2_ESM.docx]

**Supplementary Table 1.** Strains used for genome size measurement and copy number quantification, site of isolation and name of isolator.

| **Species** | **Strain** | **Origin** | **Isolator** |
| --- | --- | --- | --- |
| *A. pacificum* | ACSHO2 | Sydney Harbour, NSW, Australia | Unknown |
|  | CAWD44 | Tauranga, New Zealand | L. Mackenzie/J. Adamson |
|  | ACTRA02 | Triabunna, Tasmania, Australia | C. Bolch |
|  | CS798 | Port River, Adelaide, South Australia | N. Parker |
|  | CS315 | Port Phillip Bay, Victoria, Australia | S. Blackburn |
|  | ACCC01 | Cowan Creek, New South Wales, Australia | Unknown |
|  | CS300/01 | Samchonpo, Korea | C. Bolch |
|  | CS314/8 | Port Phillip Bay, Victoria, Australia | Ø. Moestrup |
|  | CS313/1 | Port Phillip Bay, Victoria, Australia | S. Blackburn |
|  | CS316/3 | Ballast water, Kashima, Japan | Unknown |
|  | CS319 | Ballast water “Golden Crux”, Singapore | Unknown |
| *A. catenella* | STH-M | St Helen, Tasmania, Australia | C. Bolch |
|  | AF1531 | Unknown | C. Bolch |
|  | TRIA-F | Triabunna, Tasmania, Australia | C. Bolch |
|  | STH1608 | St Helen, Tasmania, Australia | C. Bolch |
|  | ATTR/F | Triabunna, Tasmania, Australia | C. Bolch |
|  | STH-D | St Helen, Tasmania, Australia | C. Bolch |
|  | STH1604 | St Helen, Tasmania, Australia | C. Bolch |
|  | STH-F | St Helen, Tasmania, Australia | C. Bolch |
|  | STH-A | St Helen, Tasmania, Australia | C. Bolch |
|  | AF1532 | Unknown | C. Bolch |
|  | STH1625 | St Helen, Tasmania, Australia | C. Bolch |
|  | ATMP7E8 | Salt Pond, Massachussets, USA | Unknown |
|  | TRIA-E | Triabunna, Tasmania, Australia | C. Bolch |
| *A.australiense* | ATCJ33 | Cape Jaffa, South Australia, Australia | M. de Salas |
|  | ATNWB01 | North West Bay, Tasmania, Australia | C. Bolch |
|  | AABP-B | Tasmania, Australia | C. Bolch |
|  | AADVN-1 | Tasmania, Australia | C. Bolch |
|  | AT-YC-H | York Cove, Tasmania, Australia | C. Bolch |
| *A.minutum* | RCC4873(1252) | Iroise Sea, France | L. Guillou |
|  | CCMI1002 | Bantry Bay, Ireland | Unknown |
|  | RCC4877 | Iroise Sea, France | L. Guillou |
|  | CS324 | Port River, Adelaide, South Australia | S. Blackburn/J. Cannon |
|  | De1231 | Unknown, France | L. Guillou |
|  | De1699 | Unknown, France | L. Guillou |
|  | De1215 | Unknown, France | L. Guillou |
